# Environmental drivers of biogeography and community structure in a Mid-Atlantic estuary

**DOI:** 10.1007/s00442-023-05500-z

**Published:** 2024-02-14

**Authors:** Haley A. Oleynik, Joseph J. Bizzarro, Edward A. Hale, Aaron B. Carlisle

**Affiliations:** 1 School of Marine Science and Policy, College of Earth, Ocean and Environment, University of Delaware, 700 Pilottown Road, Lewes, DE 19958 USA; 2 Fisheries Collaborative Program, Cooperative Institute for Marine Ecosystems and Climate, University of California, Santa Cruz and Fisheries Ecology Division Southwest Fisheries Science Center, National Marine Fisheries Service, 110 McAllister Way, Santa Cruz, CA 95060 USA; 3 Delaware Sea Grant, School of Marine Science and Policy, College of Earth, Ocean and Environment, University of Delaware, 700 Pilottown Road, Lewes, DE 19958 USA

**Keywords:** Fish ecology, Species diversity, Species richness, Environmental drivers, Community ecology

## Abstract

Estuaries include some of the most productive yet anthropogenically impacted marine ecosystems on the planet, and provide critical habitat to many ecologically and economically important marine species. In order to elucidate ecological function in estuaries, we must understand what factors drive community dynamics. Delaware Bay is the third largest estuary in the United States and hosts over 200 species of migrant and resident fishes and invertebrates. The Delaware Division of Fish and Wildlife has conducted two long-term trawl surveys at monthly intervals in Delaware Bay since 1966. The two surveys collect data on environmental conditions, species composition, and number of fishes and macroinvertebrates across different size classes and life histories. Using a suite of multivariate approaches including hierarchical cluster analysis, canonical correlation analysis, and permutational multivariate analysis of variance, we characterized the fish and macroinvertebrate community in Delaware Bay and found that community composition and environmental conditions varied across spatial and seasonal scales. We identified four distinct biogeographic regions, based on environmental conditions and community composition, which were consistent across surveys. We found that the community was driven primarily by gradients in temperature and salinity and that abundant, frequently occurring species in the Bay have well-defined environmental associations. Our work represents the first attempt to use an existing historical survey to better understand how environmental parameters influence diversity and distribution of macrofauna within Delaware Bay, providing insight into how abiotic variables, influenced by climate, may impact the Delaware Bay ecosystem and similar estuarine ecosystems worldwide.

**Supplementary Information:**

The online version contains supplementary material available at 10.1007/s00442-023-05500-z.

## Introduction

Estuarine ecosystems provide critical ecosystem services to residents and economic value to coastal economies (e.g. Lellis-Dibble et al. 2008), yet they are among the most anthropogenically impacted aquatic ecosystems globally (Edgar et al. 2000), with temperatures rising more rapidly than other aquatic environments due to climate change (Scanes et al. 2020). Being at the land–sea interface, estuaries provide protection, foraging, spawning, and nursery habitat for a diversity of estuarine and marine species (Beck et al. 2001), but also experience heightened environmental variability and are in close proximity to anthropogenic activities (Cloern and Jassby 2012). As a result, estuarine-dependent marine species are impacted by local, regional, and ocean-scale processes (Feyrer et al. 2015).

As ecosystem-based fishery management (EBFM) gains traction, there is a need for more holistic ecosystem information to manage ocean resources (Pikitch et al. 2004). Complex ecosystem models, which are beginning to replace single species population assessments, have the ability to incorporate information about community dynamics and environmental drivers (Link et al. 2020). Therefore, elucidating ecosystem processes in estuaries can directly contribute to the development and parameterization of more accurate ecosystem models. Given the coupling of environmental variability and the physiological constraints of estuarine organisms, understanding patterns and processes of community dynamics along spatial and temporally relevant scales is a key first step to understanding broad-scale functioning of biological communities in estuaries, evaluating community response to environmental variability, and providing ecosystem information to establish effective management strategies.

Delaware Bay is a highly productive estuarine ecosystem that supports economically important fisheries throughout the East Coast of the United States. Although Delaware Bay is one of the largest estuaries in the United States, with extensive fish nursery habitat, large-scale biogeographic patterns and environmental associations of the fish and macroinvertebrate community remain poorly described. Most Delaware Bay macrofaunal research to date has focused on the physiology or ecology of single, economically important species such as Blue Crab (*Callinectes sapidus*), Weakfish (*Cynoscion regalis*), and Striped Bass (*Morone Saxatillis*) (Epifanio et al. 1984; Tilburg et al. 2005; Kahn and Helser 2005; Nye et al. 2008; Paperno et al. 2000; Chittenden 1971; Tupper and Able 2000). Previous multispecies research in Delaware Bay focused on characterizing fish assemblages in the tidal Delaware River or salt marsh creeks, and found that fish assemblages are influenced by spatial gradients in salinity, dissolved oxygen, and temperature (Able et al. 2001, 2009; Ribeiro et al. 2015), and that fish diversity is positively related to improved water quality (Weisberg et al. 1996). While these studies described the influence of environmental and anthropogenic conditions on fish assemblages, their spatial and temporal scope were relatively limited, and they focused primarily on juvenile fishes. Therefore, our understanding of Bay-wide community structure, across life histories, remains limited.

The Delaware Department of Natural Resources and Environmental Control Division of Fish and Wildlife (DNREC-DFW) has conducted two trawl surveys, one with a 16-foot net and one with a 30-foot net, once a month, at fixed sampling stations in Delaware Bay since 1966. While these surveys are among the longest running state surveys on the East Coast, they have never been used to explore community dynamics in one of the largest, most productive estuaries in the United States. Because estuaries provide vital habitat to transient marine species, many of which are caught in coastal and offshore fisheries, environmental conditions experienced by fishes in estuaries can have major influences on coastwide population dynamics and subsequent socio-economic implications. Understanding processes at localized, estuarine scales is, therefore, crucial to promoting climate resilient fisheries.

Using the DNREC-DFW trawl surveys, we characterized the estuarine macrofaunal community, a group of fish and macroinvertebrate species which co-occur in Delaware Bay, and identified environmental drivers of spatial and temporal distribution of this community. By using both the 16-foot and 30-foot trawl surveys, which typically catch juvenile and adult animals, respectively, our results illustrate patterns and processes across life history stages. We specifically focused on the relationships between local and regional scale environmental conditions and the community, which can be used for the implementation of multispecies and ecosystem-based fishery assessment and management and to help to better understand and predict how climate change may impact estuarine species.

## Methods

### Site description

Delaware Bay, located in the Mid-Atlantic Bight, is one of the largest estuaries in the United States, extending 213 km along its main axis, with a surface area of 1261 km^2^. Delaware Bay varies greatly biologically and physiochemically over spatial and seasonal scales (Pennock and Sharp 1986) and has one of the largest tidal freshwater prisms in the world, with a gradual salinity gradient ranging from 0.2 ppt to 31.8 ppt from Delaware River to the Atlantic Ocean (Stammermann and Piasecki 2012). The estuary is well mixed with a tidal range of approximately 2 m, a mean depth of 8 m, a max depth of 45 m, and an annual temperature range of – 2 °C to 28 °C (Bryant and Pennock 1988). Nearly, the entire estuarine perimeter of Delaware Bay is covered with salt marshes, comprising some of the most extensive salt marsh habitats in the Northeastern U.S. (Tiner 1985). These salt marsh habitats serve as nurseries for many species (Shenker and Dean 1979) and are frequented by later life stages of > 200 migrant and resident fishes and invertebrates, including several commercially and recreationally important species (Adkins 2008).

### Trawl surveys

**Fig. 1:**
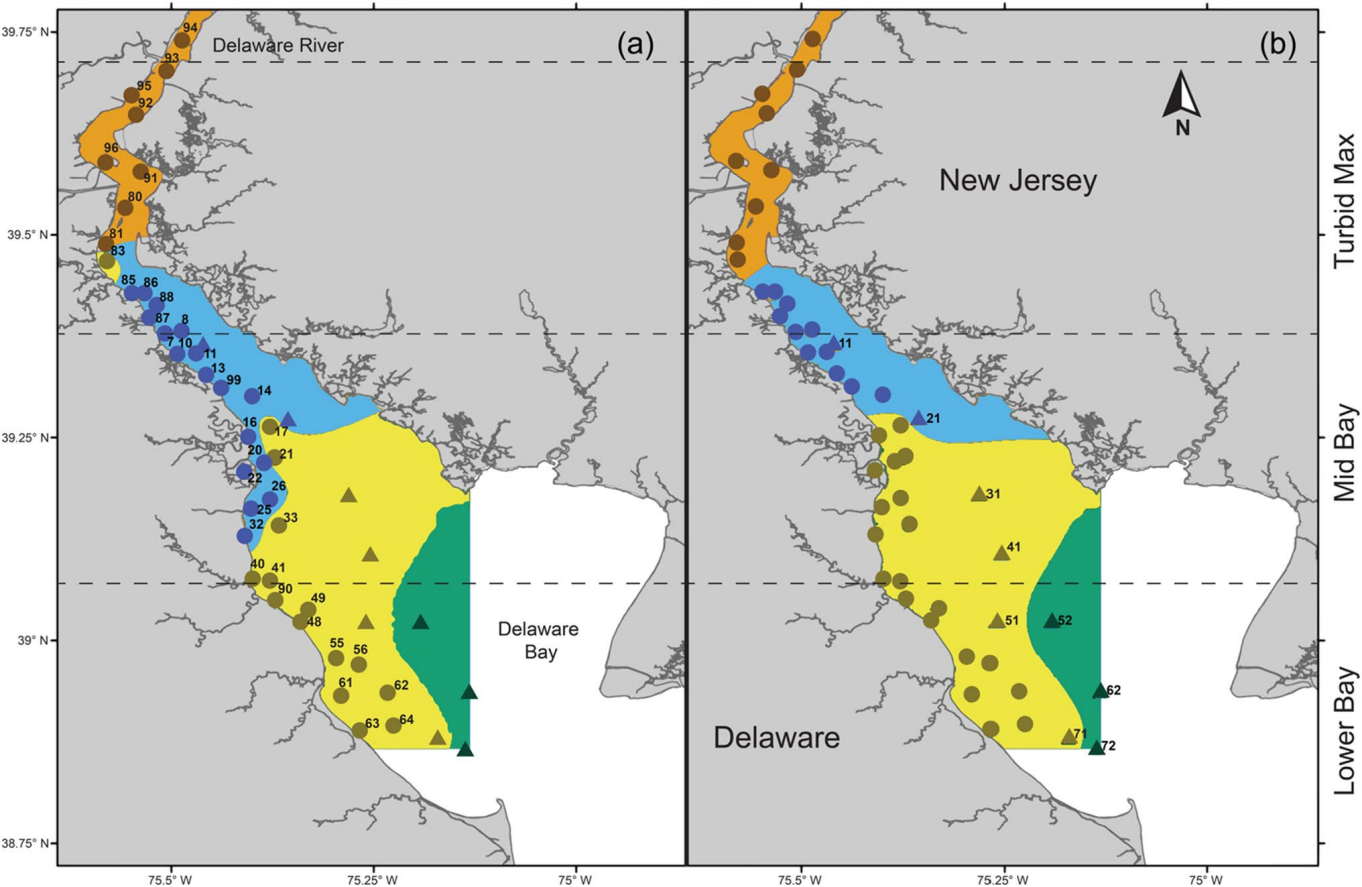
Map of Delaware Bay with regions from hierarchical cluster analysis using **a** abundances (CPUE) by species and **b** environmental variables including depth (m), temperature (°C), bottom salinity (ppt), and bottom dissolved oxygen (% saturation). Black dashed horizontal lines outline (Lower Bay, Mid Bay, Turbid Max) physiochemical regions characterized by Sharp et al. (2009). Circles represent 16-foot survey stations and triangles represent 30-foot survey stations. Colored areas represent regions drawn from cluster results using Ordinary Kriging using a spherical semi-variogram model. Orange = region 1 (Delaware River), blue = region 2 (upper bay), yellow = region 3 (mid bay), green = region 4 (lower bay)

Catch data for fish and macroinvertebrate species in Delaware Bay were obtained from two mid-water trawl surveys conducted by DNREC-DFW. A 30-foot survey, has been conducted intermittently from 1966 to 1990 and monthly from 1990 to present, excluding the months of January and February, at 9 fixed stations in the middle of the Bay, and targeted larger animals and size classes (Fig. 1). Fish species were recorded from 1966 to present, and invertebrate species were added in 1990. The 30-foot survey has a trawl net with a 32-foot (9.75 m) headrope, 42-foot footrope (12.80 m), 3-inch (7.62 cm) stretch mesh, and 2-inch (5.08 cm) mesh codend. A 16-foot survey, began in 1978 and has been conducted once monthly from April to October at 40 fixed near shore stations in Delaware Bay and the Delaware River, and targeted smaller animals and age classes. This survey began as a monthly Blue Crab survey in 1978 and added fish species to the record in 1980. The 16-foot survey net has a 16-foot (4.88 m) headrope, 21-foot (6.40 m) footrope, 1.5-inch (3.81 cm) stretch mesh, and a 0.5-inch (1.27) mesh codend.

For both trawl surveys, odometer readings from a global positioning system (GPS) unit were used to determine distance towed in nautical miles. To standardize catch data, catch per unit effort (CPUE) was calculated simply by dividing the number of individuals caught in each tow by tow distance in nautical miles. For each tow, species richness (number of species) was tallied, and the Shannon Diversity Index (*H*) was calculated to measure species diversity. The Shannon Diversity Index incorporates species richness and the relative abundance, or evenness, of each species in a given community (Krebs 1999). We chose this index because it is typically used for large communities in which the total number of species is unknown. The Shannon Diversity Index equation is shown below, where* p*_*i*_ is the proportion of species *i*, and *S*, is the number of species so that $\sum\nolimits_{i = 1}^{S} {p_{i}^{2} = 1}$, and *b* is the base of the logarithm. We calculated the index using the ‘diversity’ function from the ‘vegan’ package (Oksanen et al. 2020) in R (R Core Team 2021; version 3.6.1).

$$H = - \mathop \sum \limits_{i = 1}^{S} p_{i} log_{b} p_{i}$$

### Environmental metrics

In addition to catch information, both trawl surveys also collect abiotic data. Mean water depth was determined from fathometer readings taken at 5-min intervals from the start to end points of each tow. Both surface and bottom temperature (°C), dissolved oxygen (parts per million), and salinity (parts per thousand) were measured at the conclusion of each tow throughout the time series. We used bottom measurements of temperature, salinity, and dissolved oxygen in our analyses of the 30-foot survey, whereas surface measurements of these parameters were used for our 16-foot survey analyses. The differential use between surveys of these environmental measurements reflected the completeness of the data in each dataset, i.e. the 30-foot survey had more data on bottom measurements and the 16-foot survey had more data on surface measurements. However, because Delaware Bay is relatively shallow throughout with little stratification (Sharp et al. 2009), surface and bottom measurements for both surveys are similar. We converted dissolved oxygen to percent air saturation using the measurements (in parts per million) and corresponding salinity and temperatures. We also used indices for two longer term regional climatological conditions, the Atlantic Multidecadal Oscillation (AMO) and the North Atlantic Oscillation (NAO). The AMO is a 65-to-80-year climate cycle in the North Atlantic driven by variability in ocean thermohaline circulation (Sutton and Hodson 2005) which has been previously linked to fluctuations in fish abundance of a number of marine species (Faillettaz et al. 2019; McLean et al. 2018; Mathews et al. 2022). The NAO, another major source of interannual oceanic variability, represents the sea-level pressure difference between Iceland and the Azores, and has been linked to increased sciaenid abundance (Mathews et al. 2022) and increased CPUE of tunas (Báez et al. 2011). Data for these oscillations were obtained from the National Oceanic and Atmospheric Administration’s Earth System Research Laboratory (https://www.esrl.noaa.gov/psd/data/climateindices/).

### Statistical analyses

We used hierarchical cluster analysis (Bridges 1966) to characterize broad-scale biogeographic patterns in Delaware Bay by grouping the fixed sampling stations for each survey by environmental conditions and CPUE of each species. Hierarchical cluster analysis produces a quantitative, hierarchical classification of the dissimilarity among species or environmental data (Kulbicki et al. 2013). To explore environmental spatial variability, we clustered stations in both surveys by environmental conditions including log transformed mean temperature (°C), mean salinity (ppt), mean dissolved oxygen (% saturation), and mean depth (meters) for the entire dataset (1966–2019 for the 30-foot, and 1978–2019 for the 16-foot survey) using Euclidean distance. To explore species biogeography, we normalized CPUE for each species by removing species that occurred in less than 10% of the sampled stations to reduce the effect of rare species. After normalization, standardized CPUE from 139 species for the 30-foot and 115 species for the 16-foot survey were used to cluster the fixed sampling stations. The species cluster analysis was conducted using a Bray–Curtis similarity matrix to remove the effect of joint absences (Bray and Curtis 1957). To perform all cluster analyses, we used the Ward agglomerative clustering method (Ward 1963) which is based on the linear model criterion of least squares and is useful for large ecological datasets as it reduces the number of clusters composed of only one location and minimizes total within-cluster variation (Kulbicki et al. 2013).

The cluster analyses were carried out with the ‘hclust’ function in the ‘stats’ package in R. We determined the optimal number of clusters (i.e., groups of stations that are most similar based on environmental or species information) by examining a scree plot of the within group sum of squares vs. the number of clusters for the overall data set. Clusters were then mapped as biogeographic regions using Ordinary Kriging using a spherical semi-variogram model, implemented in ArcGIS 10.8.1 (ESRI Inc.).

Permutational multivariate analysis of variance (PERMANOVA; Anderson 2001) was used to investigate the relationship between species CPUE and external factors. PERMANOVA assesses between-group dissimilarities over within-group dissimilarities by computation of pseudo-*F* values (MS_between_/MS_within_) and permutational assessment of *P-*value (Anderson 2001) to test multivariate effects of factors on community composition given multiple species abundances. A larger relative *F* value means that a particular variable explains a greater proportion of the variation in the species abundance data. CPUE for each species in each tow were normalized by removing species that occurred in less than 2% of tows to reduce the effect of rare species. Response variables for the final PERMANOVA models included 43 species for the 30-foot and 40 species for the 16-foot survey. Temperature (°C), salinity (ppt), dissolved oxygen (% saturation), depth (meters), climate indices (NAO, AMO), spatial variables (latitude, longitude, station, and distance from the mouth of the Bay in meters), and an interaction between temperature and salinity for the 30-foot survey analysis, were used as explanatory variables. To account for temporal patterns, month and year were also included as explanatory variables. Temperature, salinity, dissolved oxygen, and depth underwent a continuity correction of + 1 to adjust for zero values and were then log transformed. For the PERMANOVA analysis, only data from 1990 to 2019 were used due to sampling inconsistencies. We used a forward stepping process where each explanatory variable was first run separately in a series of individual models to determine significance and to order variables by descending *F* value in the final model.

The PERMANOVA was conducted using the ‘adonis’ function in the ‘vegan’ package in R with 9,999 random permutations. Highly correlated variables (*r* > 0.60, *P* < 0.001) were removed, and the variable with a higher effect in the individual PERMANOVA model was chosen. For instance, all spatial measures (latitude, longitude, station, and distance from mouth of Bay) were excluded from both survey models because of their strong correlation to salinity.

When PERMANOVA results indicated a significant influence of an environmental condition on the species data, canonical correlation analysis (CCA; Hotelling 1936) was used to identify species corresponding to those environmental conditions. CCA is a constrained ordination data reduction method which seeks to identify and quantify the associations between two sets of variables. CCA provides site scores that can be used to visualize multivariate patterns and species scores to evaluate the relative contributions of individual species (Hotelling 1936). The analysis was performed with the ‘cca’ function in the ‘vegan’ package in R. We used the same normalized species data as were used in the PERMANOVA.

To further understand how the top 10 most frequently occurring species captured in each survey associated with their environment, we used conditional density plots. Presence or absence of each of these 10 species was used to assess dynamism in their probability of occurrence over a range of temperature, salinity, and dissolved oxygen values. Conditional densities describe how the conditional distribution of a categorical variable changes relative to a continuous variable. We performed this analysis using the ‘cdplot’ function in the ‘graphics’ package in R, which uses a smoother so probabilities can be viewed on a continuous scale. Because the conditional density estimation is more reliable when there are more observations at a given environmental condition, temperature data were trimmed to 5–27 °C, salinity data to 0–30 ppt, and dissolved oxygen data to 70–100% saturation.

## Results

Nearly 200 species were caught in the two Delaware Bay trawl surveys over the past six decades. In the 30-foot survey, a total of 871,771 individuals from 139 species were caught in 3,260 tows during the 43 years between 1966 and 2019. A mean of 267 (standard deviation ± 499) individual animals and 7.9 (± 3.4) distinct species were caught in each tow, resulting in a mean diversity estimate of 1.13 (± 0.48). In the 16-foot survey, a total of 4,245,102 individuals from 166 species were caught in 10,402 tows during the 41 years between 1978 and 2019. A mean of 408 (± 705) individuals and 7.0 (± 3.1) species were caught in each tow, resulting in a lower mean diversity estimate (0.92 ± 0.46). In both surveys, across time, a small number of species made up a large proportion of the catch (Table 1). In the 30-foot survey, Weakfish and Hogchoker (*Trinectes maculatus*) were the most abundant species caught, composing 32.8% and 10.4% of the numerical catch, respectively. In the 16-foot survey, Blue Crab and Bay Anchovy (*Anchoa mitchilli*) were the most abundant species, making up 29% and 25.3% of the catch, respectively.

**Table 1:** Catch statistics and ecological information of key species

Common Name	Scientific Name	Distribution	Habitat	Feeding Habit	30-foot Survey				16-foot Survey			
					Number	Percent	%-Months	Regions	Number	Percent	%-Months	Regions
Weakfish	*Cynoscion regalis*	ST	D	Pr	28,5891	32.8	79	2,1,3	55,5915	13.1	87	3,2,1
Hogchoker	*Trinectes maculatus*	ST	D	Pr	9,0861	10.4	97	1,2,3	30,8943	7.3	94	1,2,3
Spot	*Leiostomus xanthurus*	ST	D	Pr	7,8414	9.0	53	2,1,3	12,7264	3.0	76	2,1,3
Atlantic Croaker	*Micropogonias undulatus*	ST	D	Pr	7,4364	8.5	49	2,1,3	47,3620	11.2	63	1,2,3
Scup	*Stenotomus chrysops*	ST	D	Pr	6,9595	8.0	50	3,2	4444	0.1	30	2,3
Butterfish	*Peprilus triacanthus*	ST	BP	Pr	3,1336	3.6	70	3,2,1	2388	0.1	58	3,2,1
Blue Crab	*Callinectes sapidus*	ST	B	Pr	3,0255	3.5	65	1,2,3	1,23,3598	29.1	99	2,1,3
White Perch	*Morone americana*	T	D	Pr	2,9318	3.4	40	1,2	15,2927	3.6	87	1,2,3
Smooth Dogfish	*Mustelus canis*	ST	D	Pr	2,5147	2.9	65	3,2,1	1465	0.0	59	3,2,1
Spotted Hake	*Urophycis regia*	ST	D	Pr	2,1883	2.5	82	1,2,3	10,1626	2.4	59	3,2,1
Windowpane Flounder	*Scophthalmus aquosus*	T	D	Pr	1,6311	1.9	94	3,2,1	5091	0.1	63	3,2,1
Atlantic Menhaden	*Brevoortia tyrannus*	ST	P	Pl	8682	1.0	71	2,3,1	7375	0.2	77	2,3,1
Oyster Toadfish	*Opsanus tau*	ST	RA	Pr	8082	0.9	76	1,2,3	4129	0.1	89	3,2,1
Squid	*Squid spp.*	ST/T	P	Pr	7946	0.9	50	3	1846	0.0	32	3
Bay Anchovy	*Anchoa mitchilli*	ST	P	Var	7634	0.9	38	3,2,1	1,07,2841	25.3	93	3,2,1
Northern Searobin	*Prionotus carolinus*	T	D	Pr	7246	0.8	50	3,2,1	3346	0.1	59	3,2,1
Clearnose Skate	*Raja eglanteria*	ST	D	Pr	5030	0.6	63	3,2	297	0.0	29	
Striped Bass	*Morone saxatilis*	T	D	Pr	4770	0.6	52	1,2,3	3,2304	0.8	75	1,2,3
Summer Flounder	*Paralichthys dentatus*	T	D	P	4733	0.5	83	2,1,3	2841	0.1	89	3,2,1
Atlantic Herring	*Clupea harengus*	T	BP	Pl	4633	0.5	27	3,2,1	4814	0.1	18	3,2,1
Bullnose Ray	*Myliobatis freminvillei*	ST	BP	Pr	3741	0.4	38	3,2	67	0.0	14	3
Black Drum	*Pogonias cromis*	ST	D	Pr	3184	0.4	30	1,2,3	1568	0.0	26	2,1,3
Silver Perch	*Bairdiella chrysoura*	ST	D	Var	3128	0.4	29	1,2	5078	0.1	54	3,2,1
Northern Kingfish	*Menticirrhus saxatilis*	ST	D	Pr	3112	0.4	44	2,3,1	3768	0.1	44	3,2,1
Red Hake	*Urophycis chuss*	T	D	Var	3090	0.4	28	3,2,1	2975	0.1	20	3
Striped Searobin	*Prionotus evolans*	T	RA	Pr	2649	0.3	48	3,2,1	1845	0.0	47	
Alewife	*Alosa psuedoharengus*	T	P	Pr	2626	0.3	45	2,1,3	3324	0.1	52	1,2,3
Northern Puffer	*Sphoeroides maculatus*	T	D	Pr	2483	0.3	32	3	231	0.0	29	
Spiny Dogfish	*Squalus acanthias*	T	B	Pr	2457	0.3	31	3,2	4	0.0	1	3
Silver Hake	*Merluccius bilinearis*	T	D	Pr	2320	0.3	28	3	51	0.0	5	3
Horseshoe Crab	*Limulus polyphemus*	ST	B	Var	2066	0.2	41	3,2,1	2,4125	0.6	70	3,2
Little Skate	*Raja erinacea*	T	D	Pr	1779	0.2	39	3,2	74	0.0	9	3
Hickory Shad	*Alosa mediocris*	T	P	Pr	1582	0.2	44	2,3	101	0.0	16	
Atlantic Moonfish	*Selene setapinnus*	ST	BP	Pr	1546	0.2	14	2	135	0.0	16	
Bluefish	*Pomatomus saltatrix*	ST	P	Pr	1242	0.1	44	3,2	1194	0.0	56	
American Shad	*Alosa sapidissima*	ST	P	Pr	944	0.1	34	3,1,2	1207	0.0	35	
Eutropus Flatfishes	*Etropus spp.*	ST	D	Pr	684	0.1	43	3	626	0.0	42	3
Winter Flounder	*Pleuronectes americanus*	T	D	Pr	578	0.1	20	3	492	0.0	27	
Black Seabass	*Centropristis striata*	T	RA	Pr	570	0.1	27	3,2	1111	0.0	63	
Bluntnose Stingray	*Dasyatis say*	ST	D	Pr	560	0.1	26	2,3	52	0.0	11	3
Striped Anchovy	*Anchoa hepsetus*	ST	P	Pl	499	0.1	12	3,2	2779	0.1	37	3,2,1
Sandbar Shark	*Carcharhinus plumbeus*	ST	BO	Pr	295	0.0	21	2	29	0.0	8	
Winter Skate	*Raja ocellata*	T	D	Pr	207	0.0	14	3	2	0.0	1	
Crevalle Jack	*Caranx hippos*	ST	RA	Pr	162	0.0	10		268	0.0	17	
Northern Stargazer	*Astroscopus japonicus*	T	D	Pr	83	0.0	10	1	336	0.0	38	1
Naked Goby	*Gobiosoma bosc*	TR	D	Pr	76	0.0	2		1207	0.0	57	
Striped Cusk-eel	*Ophidion marginatum*	T	D	Pr	53	0.0	8		2,7130	0.6	90	2,3,1
Blackcheek Tonguefish	*Symphurus plagiusa*	ST	D	Pr	39	0.0	4	7,7,2	0.0	15	1	
American Eel	*Anguilla rostrata*	ST	D	Pr	25	0.0	5	1,0,2,8,8	0.2	93	12	
White Catfish	*Ameiurus catus*	ST	D	Pr	21	0.0	3	4,6,1	0.0	43	1	
Northern Pipefish	*Sygnathus fuscus*	ST	D	Pr	17	0.0	4	4,3,1,7	0.1	91	321	
Channel Catfish	*Ictalurus punctatus*	ST	D	Var	16	0.0	3	1,6,0,9,8	0.4	82	1	
Brown Bullhead	*Ameiurus nebulosus*	ST	D	Pr	0	0.0	0	7,1,4	0.0	44	1	

Distribution (ST = subtropical, T = temperate, TR = tropical), habitat (D = demersal, BP = benthopelagic, RA = reef associated, B = benthic, P = pelagic), feeding habit (Pr = predator, Pl = planktivore, Var = variable) from FishBase (Froese and Pauly 2021), total number of animals across all tows, percent of numerical catch (percent), percent of months caught, and regions species are found in (see Fig. 1)

**Fig. 2:**
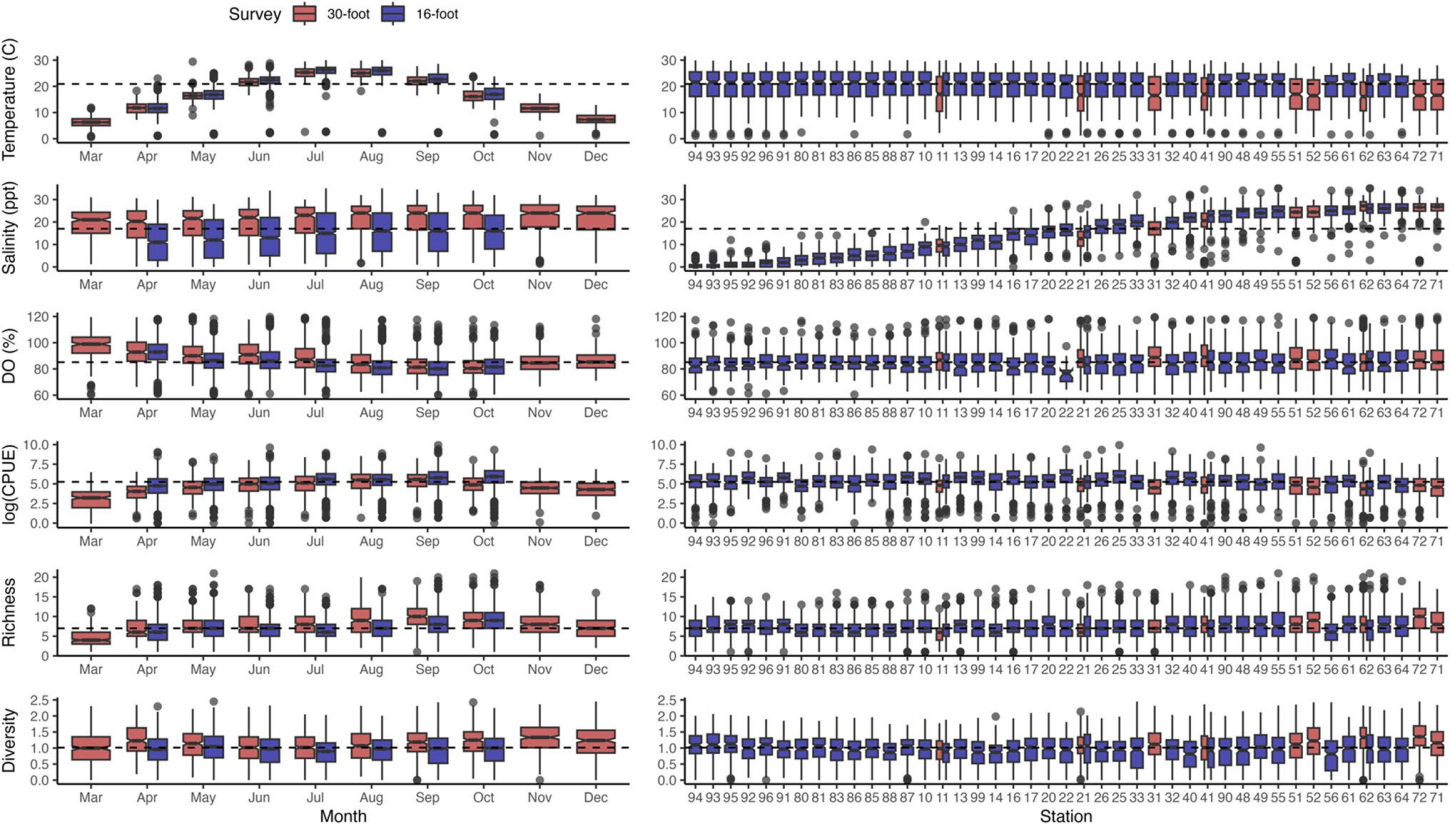
Average temperature (°C), salinity (parts per thousand), dissolved oxygen (% saturation), log transformed CPUE (number of individuals per distance towed), species richness, and species diversity by month and station across all years for the 30-foot (in red) and 16-foot (in blue) surveys. Dashed horizontal lines represent across-year median values

Both the community in Delaware Bay and the measured environmental conditions from the surveys varied spatially and temporally (Fig. 2, Supplementary Table 1). Highest catch rates in the 30-foot survey were observed in July, August, and September, highest species richness in September, and highest species diversity in April and November. Highest catch rates and species richness in the 16-foot survey were observed in September and October and highest species diversity was observed in May and October. Spatially, catch rates were consistent across all stations, but species richness and species diversity increased closer to the mouth of the Bay (Fig. 2). For both surveys, temperature and dissolved oxygen were relatively constant across space, but salinity varied greatly, increasing toward the mouth of Delaware Bay. Temperatures were generally higher in summer months, and dissolved oxygen was highest in early spring and late fall (Fig. 2).

**Fig. 3:**
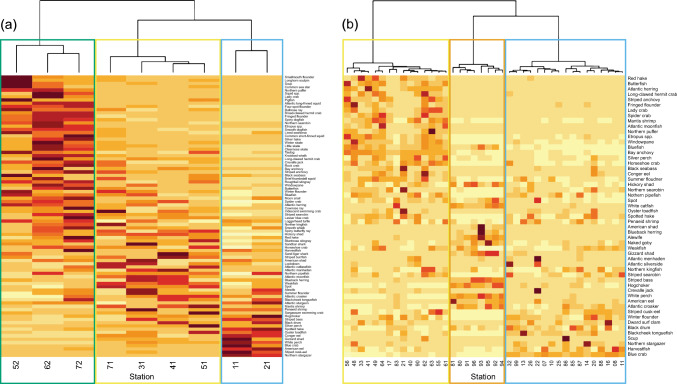
Heatmap of species hierarchical cluster results for the **a** 30-foot and **b** 16-foot survey for species caught in at least 50% of the fixed sampling stations. Darker colors represent higher abundances, lighter colors represent lower. Outline colors correspond to regions in Fig. 1

For both surveys, results of hierarchical cluster analysis using CPUE by species and mean environmental conditions yielded three primary clusters (Fig. 3; Supplementary Figure 4). Because the sampled area of the two surveys did not completely overlap, we mapped clusters, resulting from the cluster analysis, as four distinct biogeographic regions. The upper region, in red, was only covered by the 16-foot survey (Fig. 1), was characterized by low salinity, with a mean of 2.37 ppt (± 1.36), and comprised species that inhabit lower salinity and freshwater environments (Fig. 3). The next region, in yellow, included the furthest north stations in the 30-foot survey, along with some stations from the 16-foot survey, and was characterized by a mean salinity of 9.50 ppt (± 3.10). The third region, in green, consisted of the furthest south stations in the 16-foot survey and the majority of stations in the 30-foot survey and was characterized by a mean salinity of 21.61 ppt (± 4.22). The lower region closest to the mouth of the Bay, in blue, was only covered by the 30-foot survey and was characterized by high salinity, with a mean of 27.77 ppt (± 1.14), the greatest depth, and comprised more marine-associated species.

We found that localized environmental conditions have a strong effect on the marine community in Delaware Bay based on results of the PERMANOVA. Bottom temperature, bottom salinity, depth, month, year, dissolved oxygen, the AMO, the NAO, and the interaction between bottom temperature and salinity were significant explanatory variables for species composition based on catch rate for the 30-foot survey (Table 2). Temperature had the greatest pseudo-*F* value, indicating it has the strongest effect on species composition among explanatory variables, followed by salinity (Table 2). For the 16-foot survey, surface salinity, surface temperature, depth, month, dissolved oxygen, the AMO, and year were significant explanatory variables (*P* < 0.01) for species composition based on catch rate, with salinity having the highest pseudo-*F* value (Table 2). The magnitude of the effect of both temperature and salinity was generally higher in the 16-foot survey.

**Table 2:** PERMANOVA model of species CPUE among environmental, climate, and temporal variables

Variable(s)	*F*	df	r2	*P*
30-foot survey				
Temperature (°C)	162.04	1	0.070	0.0001
Salinity (ppt)	65.59	1	0.028	0.0001
Depth	26.45	1	0.012	0.0001
Month	14.17	9	0.055	0.0001
AMO	12.71	1	0.006	0.0001
Year	3.86	23	0.039	0.0001
Dissolved oxygen (%)	2.97	1	0.001	0.0008
NAO	2.32	1	0.001	0.0044
Temp × salinity	20.83	1	0.009	0.0001
Residuals		1799	0.779	
16-foot survey				
Salinity	480.66	1	0.104	0.0001
Temperature	304.77	1	0.066	0.0001
Month	50.82	6	0.066	0.0001
Depth	29.13	1	0.006	0.0001
Year	27.27	12	0.070	0.0001
Dissolved oxygen	16.45	1	0.004	0.0001
NAO	6.96	1	0.002	0.0001
AMO	8.01	1	0.002	0.0001
Salinity × temp	48.66	1	0.010	0.0001
Residuals		3116	0.671	

*F*-statistic, degrees of freedom (df), amount of variability explained (r2), and *P*-value

**Fig. 4:**
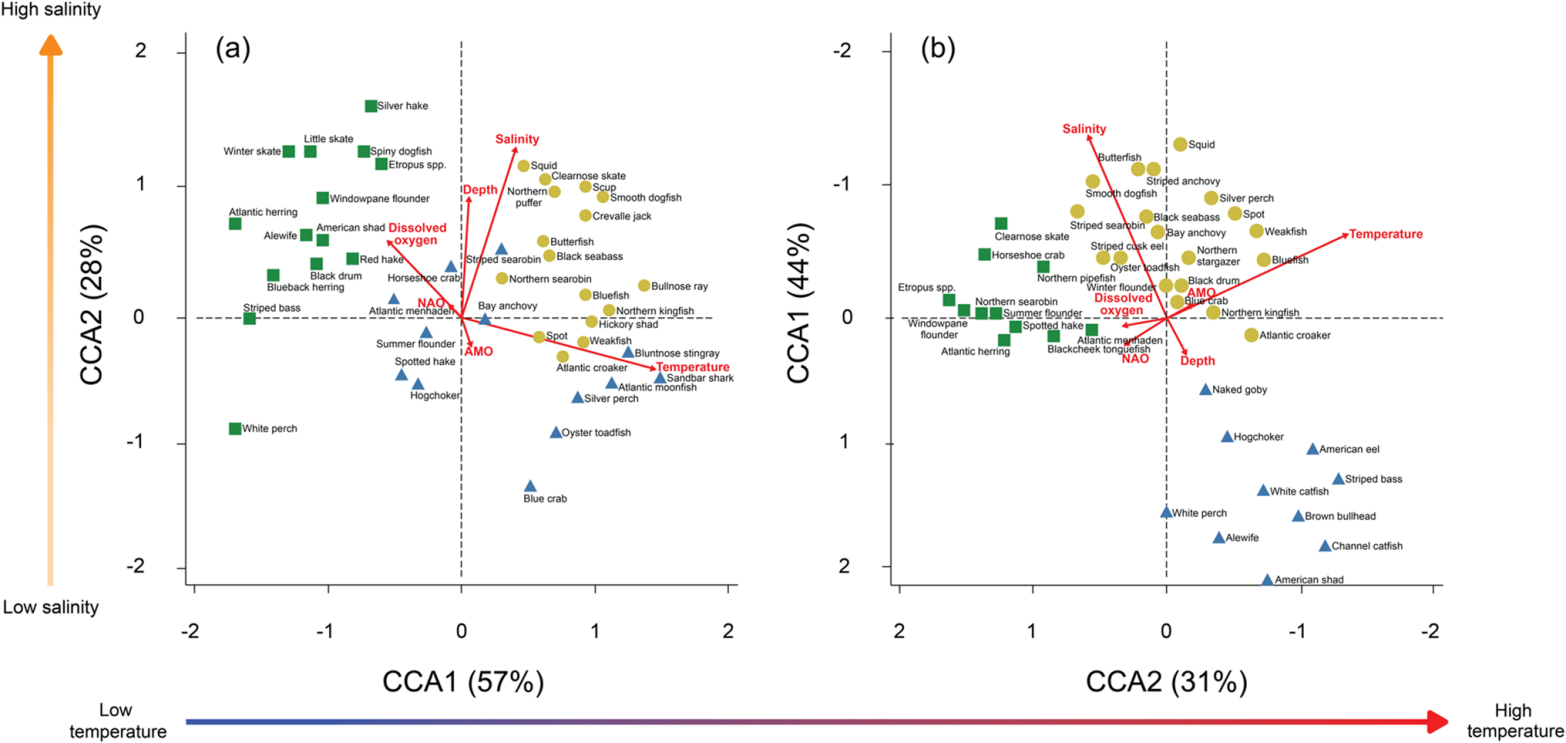
CCA biplot of the relationship between environmental variables and species for the **a** 30-foot survey and **b** 16-foot survey. 16-foot survey axes are flipped to visualize analogous temperature and salinity gradients between surveys. Arrows represent environmental variables and climate indices. The *X* axis is explained primarily by temperature increasing in the positive direction and dissolved oxygen increasing in the negative direction. The *Y* axis is explained primarily by salinity increasing in the positive direction. Results of a hierarchical cluster analysis using the loadings on the CCA axes are represented with shapes and colors of species. Note: colors do not correspond to the spatial cluster analysis in Fig. 1

The Delaware Bay marine community varied considerably with changes in temperature and salinity based on the results of the CCA, which were consistent with those of the PERMANOVA (Fig. 4). The 30-foot survey CCA model was significant (*F* = 22.10, *P* = 0.001) and explained 6.75% of the variance in the data set, with the first two axes accounting for 81.2% of the total (CCA1: *F* = 75.34, *P* = 0.001, 56.7%, CCA2: *F* = 32.32, *P* = 0.001, 24.4%). All input variables except the NAO were significant (*P* < 0.001). The first axis can be described primarily by bottom temperature (loading = 0.96) and bottom dissolved oxygen (loading = -0.37). The second axis reflects differences in bottom salinity (loading = 0.85) and depth (loading = 0.61). The 16-foot survey CCA model was also significant (*F* = 51.79, *P* = 0.001) and explained 9.02% of the variance in the data set, with the first two axes accounting for 90.0% of the total (CCA1: *F* = 184.34, *P* = 0.001, 59.3%, CCA2: *F* = 95.46, *P* = 0.001, 30.7%). All input variables, except the NAO, were significant (*P* < 0.001). The first axis can be described by changes in surface salinity (loading = − 0.92). The second axis can be described primarily by surface temperature (loading = − 0.90). For both surveys, the magnitude of these loadings on the axes are consistent with the results of the PERMANOVA.

By clustering the CCA loadings, we describe guilds of species that associate with similar environmental conditions. These guilds can illustrate what impact warming might have on the estuarine community in Delaware Bay. The cluster represented by green squares in both CCA biplots comprises species that occur at lower temperatures and slightly higher salinities (Fig. 4). Alternatively, species that, fall on the opposite end of the temperature defined CCA axis, shown in both blue triangles and yellow circles in the CCA biplots, occur at higher temperatures, with species represented by blue triangle being caught at lower salinities and species represented by yellow circles at higher salinities (Fig. 4).

**Fig. 5:**
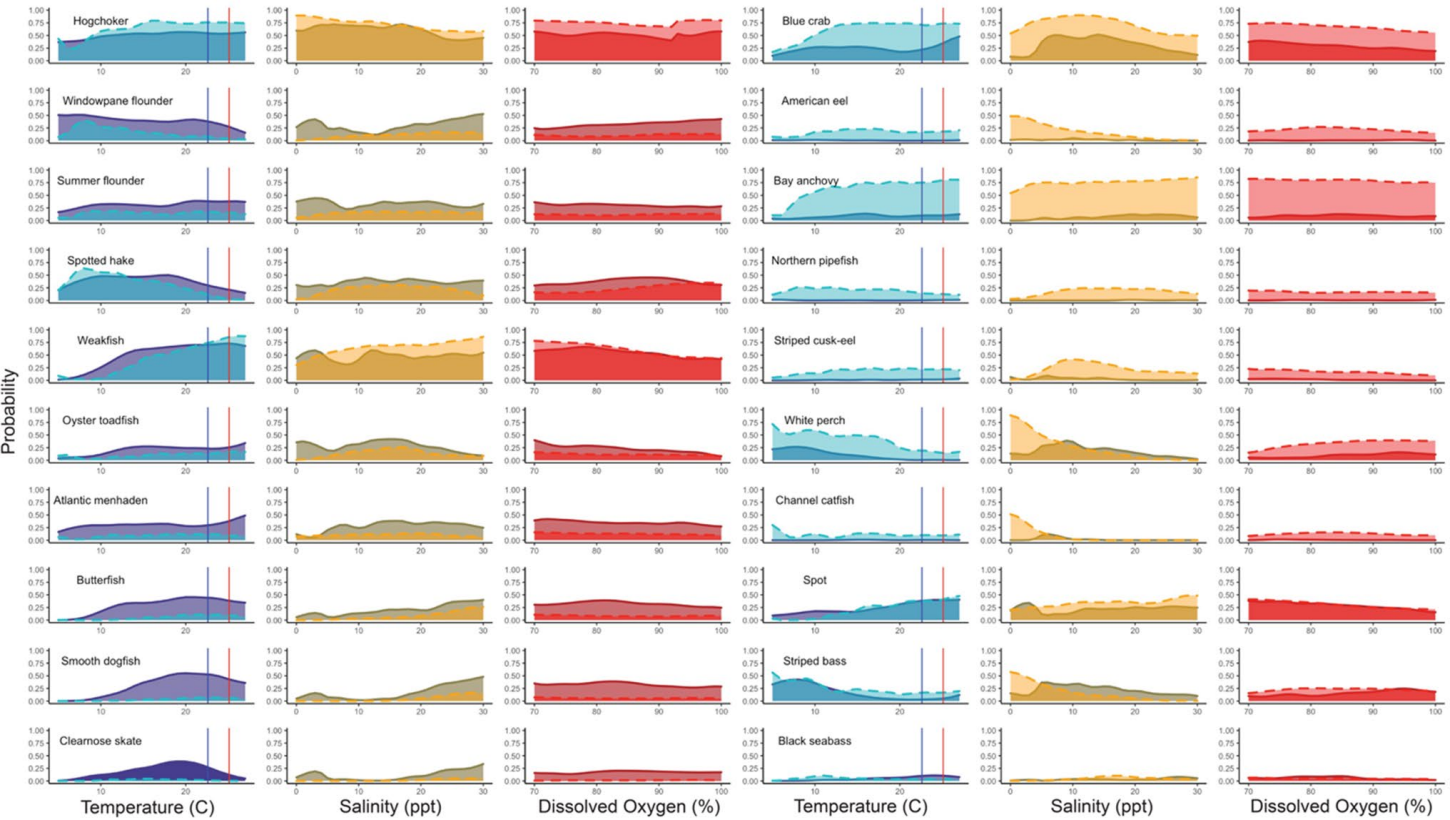
Conditional density of occurrence of top 10 most frequently occurring species in the 30-foot (left) and 16-foot (right) surveys by temperature (°C), salinity (parts per thousand), and dissolved oxygen (% saturation). Solid dark colored lines represent the 30-foot survey, dashed light colored lines represent conditional densities for those species caught in the 16-foot survey. Blue vertical line represents current mean summer temperature in Delaware Bay (22.6 °C). Red vertical line represents projected mean summer temperature in 50 years (25.1 °C; Saba et al. 2016)

Comparable with the CCA community results, abundant, frequently occurring species caught in both trawl surveys had strong associations to temperature, salinity, and dissolved oxygen. For the 30- and 16-foot surveys, the 10 most frequently occurring species were recorded in at least 60% and 75% of months sampled, respectively. The conditional density analyses revealed extremely similar patterns in species occurrence across measured environmental conditions for both surveys (Fig. 5). Results of the conditional density analyses were also consistent with species’ position on the CCA biplot and grouping in the post-hoc cluster analysis of the CCA loadings.

## Discussion

In this study, we provide a comprehensive description of the fish and macroinvertebrate community and its environmental associations, and a characterization of distinct biogeographic regions in Delaware Bay. By utilizing long-term data sets from the 16-foot trawl survey, which typically catches smaller, younger animals, and for 30’, larger, adult animals, we were able to analyze distribution and abundance of species across size classes and life history stages. Linking spatial and temporal variability in the marine environment with catch trends in multiple species across life histories provides new insight into the community ecology of Delaware Bay.

Our analyses reveal spatial variability in environmental conditions and community composition in Delaware Bay. Overall, biodiversity increases with distance from Delaware River as a function of salinity, with higher mean species richness and species diversity observed closer to the mouth of the estuary. Observed catch rate and species richness were also highest in summer and early fall when temperatures were highest and dissolved oxygen levels were lowest. Species diversity, conversely, was highest in April and November, when temperatures were lower but dissolved oxygen was relatively high. This intra-annual pattern in species diversity is likely due to the evenness component of the Shannon Diversity calculation. In spring and fall months, when catch rate is lower, the number of all species inhabiting the Bay are more even, and there is likely an overlap between summer and winter guilds, increasing the total number of species and the evenness of relative abundances, which results in higher diversity.

We identified three distinct biogeographic regions of Delaware Bay based on both environmental conditions and species guilds. Previous research has identified three major areas of the Delaware Estuary along physiochemical lines (Fig. 1; Sharp et al. 2009). Our analysis went a step beyond the Sharp et al. (2009) physical description of Delaware Bay by incorporating both physical and biological information to characterize biogeographic regions. Our results yielded three regions in the area Sharp et al. (2009) define as the ‘mid bay’ and ‘lower bay’. Differences in salinity, temperature, and dissolved oxygen did not vary substantially in these three regions, but fish guilds differed greatly, allowing a more accurate ecological definition of this area. Because the data we used do not extend to the New Jersey side of the estuary, we were unable to categorize the Southeast corner of the Bay. More data are needed to determine if this area fits into one of the biogeographic regions we describe or constitutes an entirely different region.

The fact that both surveys produced extremely similar results with respect to the biogeography of the Bay suggests these community patterns are consistent despite potential differences in age- and size-based selectivity of the two gears. The almost identical regions drawn by the environmental and species clusters for both surveys suggest species in Delaware Bay are tightly associated with their local environment and indicates that bay-wide community spatial structure is likely driven by environmental association and habitat suitability. Understanding patterns in biogeography has applications for conservation (Whittaker et al. 2005) and fisheries management (Chevallier et al. 2021). Our biogeographic characterization of regions in Delaware Bay has the potential to incorporate ecosystem information into management of marine resources and can be used to monitor single species, as it provides information about where many species inhabiting the Bay are likely to be found.

Several examples from our study can be used to examine how responses to environmental parameters drive fluctuations in the catch of individual species. For example, Blue Crab, which represent the largest inshore fishery on the East Coast, were more likely to be found at higher temperatures. For both surveys, Blue Crab fell on the higher end of the temperature determined CCA axis, and there was a greater chance of catching them at higher temperatures according to the conditional density plots. These results reflect the fact that Blue Crab abundance peaks in late summer and early fall in Delaware Bay (Kahn et al. 1988). Previous research has found that severe winters can cause major mortality in Delaware Bay Blue Crab resulting in subsequent declines in commercial landings (Kahn and Helser 2005; Rome et al. 2005), meaning the relationship Blue Crab has to temperature, reflected in both the CCA and conditional density analyses, has direct socio-economic implications for coastal communities.

Weakfish, one of the most abundant species in Delaware Bay, is also seasonally abundant, with highest abundances in summer months and generally higher juvenile abundances in lower salinity regions (Paperno et al. 2000). This, too, is reflected our CCA results. In the 30-foot survey, Weakfish were found at relatively high temperatures but moderate salinities, while in the 16-foot survey, they were found in the higher temperature and higher salinity quadrant of the CCA biplot. The conditional density analysis confirmed this association, as temperature relationships between the surveys looked nearly identical, while the salinity relationship was stronger in the 16-foot survey.

Striped Bass, another economically important species in the Mid-Atlantic region, has been studied extensively in Delaware Bay as Delaware River is one of the primary spawning areas for the coastwide Striped Bass population (Chittenden 1971). Being an anadromous species, Striped Bass spawn in rivers and later forage in estuarine habitats (Tupper and Able 2000). As they grow, Striped Bass move from fresh to saltwater, and adults typically spend little time in freshwater unless they are spawning. These life history patterns were apparent in our results. At low salinities (< 10 ppt), there was a higher chance of catching Striped Bass in the 16-foot survey, and almost no chance at high salinities (> 20 ppt) while, the probability of catching Striped Bass in the 30-foot survey was more evenly distributed across salinity levels. This interpretation is supported by the CCA analysis as Striped Bass fall in the middle of the salinity axis for the 30-foot survey, but on the extreme low end in the 16-foot survey. These results provide evidence that the surveys intercept diadromous fishes throughout periods of estuarine ingress and egress to/from the spawning/nursery grounds and further illustrate the tight associations that single species in Delaware Bay have to surrounding environmental conditions.

We found a strong relationship between the marine community and the environment (i.e., temperature and salinity) based on our PERMANOVA results, indicating that localized environmental conditions were more influential on the species captured than large-scale climate oscillations (Table 2). While the primary driver of community composition was different between the surveys, the results clearly demonstrate that temperature and salinity were the two strongest drivers of biodiversity in Delaware Bay. The difference between surveys in terms of the relative importance of both parameters was likely due to spatial coverage. Because the 16-foot survey extends into the Delaware River, where salinities are lower, the gear sampled a much wider range of salinities than the 30-foot survey.

The strength of the relationships observed between species catch rates and temperature has potentially profound implications for the future of the marine community in Delaware Bay. Fish species often respond differently, based on their species-specific physiologies and ecologies, to changes in temperature. In some cases, increased water temperatures may enhance growth or reproduction of estuarine species (Gillanders et al. 2011), while for others, warming can negatively impact performance and survival (Madeira et al. 2016). Long-term estuarine studies found that climate warming has led to shifts in community composition through time by changing population abundances of single species and the subsequent reorganization of the entire community (Gillanders et al. 2011; McLean et al. 2018; Morson et al. 2019). A study by Flanagan et al. (2019) found that changes in community composition in 160 marine fish and invertebrate communities were explained primarily by species associations with temperature, underlining the importance temperature plays in the composition of aquatic communities globally.

Surface temperatures on the Northeastern shelf of the United States have risen about 3 °C since 1970 (Kleisner et al. 2017), and Saba et al. (2016) predicted that the upper 300 m of the water column in the Northeast Atlantic will warm by 2.5 °C in 50 years. The current mean summer temperature in Delaware Bay, when highest catch rates of marine animals occur in both surveys, is 22.6 °C. Under this projected temperature change, in 50 years, temperatures could be closer to 25.1 °C, and possibly higher under the assumption that temperatures in estuaries rise at faster rates than the ocean average (Scanes et al. 2020). Based on the probability of occurrence in Delaware Bay relative to temperature as determined through our conditional density analysis, under projected temperature increases some species would be less likely to occur, while others may increase in prevalence (Fig. 5). Considering temperature is one of the strongest drivers of community in Delaware Bay, as climate change continues to drive warming, the community is likely to change correspondingly. Hare et al. (2016) suggested that diadromous fishes, like Striped bass may suffer from the onset of climate change, while coastal and pelagic species may be positively impacted by a warming climate. Data sets like the one analyzed here will become powerful tools in detecting and analyzing how these species respond in the future.

Our results indicate that Delaware Bay is composed of a diverse community of fishes and macroinvertebrates, each with species-specific environmental associations. Our spatial analysis demonstrated that there are unique species guilds inhabiting different regions of the Bay due to the strong salinity gradient. The high variability in temperature that Delaware Bay experiences throughout the year dictates local variability in temporal occurrence of species. Our CCA and conditional density analyses use the variation in temperature throughout the year to characterize species-environment associations, and the guilds described by the post hoc cluster of CCA loadings can illustrate what impact warming might have on the estuarine community in Delaware Bay. The cluster depicted by green squares in both CCA biplots represents a group of species which, under future temperature changes, may no longer find Delaware Bay suitable habitat. Some of the species found in this cluster, like American Shad (*Alosa sapidissima*) and Blueback Herring have been identified in a recent vulnerability assessment of the Northeast Shelf (Hare et al. 2016) to have a very high biological sensitivity and high climate exposure. In addition, for many species in this cluster, such as Winter Skate (*Leucoraja ocellata*), Silver Hake (*Merluccius bilinearis*), and Atlantic Herring (*Clupea harengus*), Delaware Bay is at the southern end of their species range (Froese and Pauly 2021). The species in this cluster are also defined by slightly higher loadings on the axis represented by salinity in the CCA biplot indicating they are likely to be associated with marine habitats, moving in and out of Delaware Bay, and presumably up and down the East Coast. Therefore, increased future temperatures have the potential to impact local and regional fisheries and economies.

Alternatively, species which fall on the opposite end of the temperature defined CCA axis may be more resilient to continued increasing temperatures. In both surveys, Weakfish fell in the high temperature, moderate salinity region of the CCA biplot. Research has shown that juvenile Weakfish are physiologically capable of tolerating levels of hypoxia which are common in estuaries in summer months (Stierhoff et al. 2009), and thus may be better equipped to handle increased temperatures and decreased dissolved oxygen. Other species found in this region of the CCA biplot, including Sandbar Shark (*Carcharhinus plumbeus*) and Spot (*Leiostomus xanthurus*) have ranges where Delaware Bay is in middle or northern extent of their distribution (Froese and Pauly 2021). Therefore, increased temperature, and possible range shifts, may not impact the abundance or occurrence of these species in Delaware Bay.

The data sets used in these analyses came from long-term, state conducted trawl surveys which are used to monitor and manage single species. Our results demonstrate that a surveys like this can be used to look at community dynamics and environmental links in a large estuary where these patterns have not yet been explored. This study was an important first step in analyzing this rich community data set to identify spatial and interannual patterns in the fish and macroinvertebrate community of Delaware Bay. Since these surveys has been conducted since the 1960s, it also provides a time series of these community and environmental dynamics, which we are currently exploring further to address how the community has changed over time. Government sponsored surveys conducted by other monitoring programs throughout the United States have similarly provided valuable insight into patterns and processes of aquatic communities in other regions (Jordan et al. 2010; Smith et al. 2010; Buchheister et al. 2013; Bell et al. 2014) and should continue to be used to draw links between ecological dynamics and environmental conditions, understand drivers of species abundance and distribution, and evaluate ecosystem vulnerability so that important marine resources can be conserved and managed into the future.

## Conclusion

Our study contributes to the understanding of fish community dynamics in estuarine ecosystems, essential habitats for many economically and ecologically important marine species. Being at the land-sea interface, and heavily impacted by human activity, understanding how environmental conditions influence ecological function in estuaries is critical for improving management and preparing for future environmental change. We describe the fish and macroinvertebrate community across life history stages in one of the largest estuaries in the United States, a bay that serves as a key nursery and foraging habitat for species across the Eastern seaboard. We described clear biogeographic regions of Delaware Bay and show that fish and invertebrate species in the Bay are highly influenced by the localized temperature and salinity. We also described species-specific patterns by mapping the most abundant, frequently occurring species in Delaware Bay both geographically and across environmental conditions. This information can help to assess the vulnerability of some taxa to changing environmental conditions in Delaware Bay and be used to inform future community and single species studies in Delaware Bay and in other regions that collect similar data sets across the globe.

### Supplementary Information

Below is the link to the electronic supplementary material.

Supplementary file1 (DOCX 1998 KB)

## Data Availability

The datasets used in this study are available from DNREC-DFW on reasonable request.

## Code availability

All R code used to conduct analyses in this study are available from the corresponding author upon request.
