# Supplementary material for: Environmental drivers of biogeography and community structure in a Mid-Atlantic estuary
Source: Oecologia. 2024 Feb 14;204(3):543–57. doi: 10.1007/s00442-023-05500-z (PMC10980636; doi:10.1007/s00442-023-05500-z)
Supplement: Supplementary file 1 — Supplementary file1 (DOCX 1998 KB) [file 442_2023_5500_MOESM1_ESM.docx]

<A>Supplemental Materials

**Fig 1.** Difference in a) CPUE and b) frequency of occurrence for key species between 16-foot and 30-foot survey.

**Fig 2.** Dendrograms from hierarchical cluster analysis for environmental variables (top) and species abundances (bottom) for the a) 30-foot trawl and b) 16-foot survey.

**Fig 3.** Dendrogram from hierarchical cluster of CCA loadings for a) 16-foot and b) 30-foot survey. Colored shapes correspond to those shown in Figure 4.

**Fig 4.** Log CPUE (number of individuals per area swept), species richness, and species diversity by temperature (°C), salinity (parts per thousand), dissolved oxygen (% saturation), and depth (meters). Points represent observations, lines represent LOESS curve fit with standard error. Star represents statistical significance of p<0.01 from Pearson correlation.

**Fig 5.** Scree plots for evaluation of clusters in hierarchical cluster analysis for both environmental (temperature, salinity, dissolved oxygen, and depth) measures and species abundances by station for the 30-foot and 16-foot surveys.


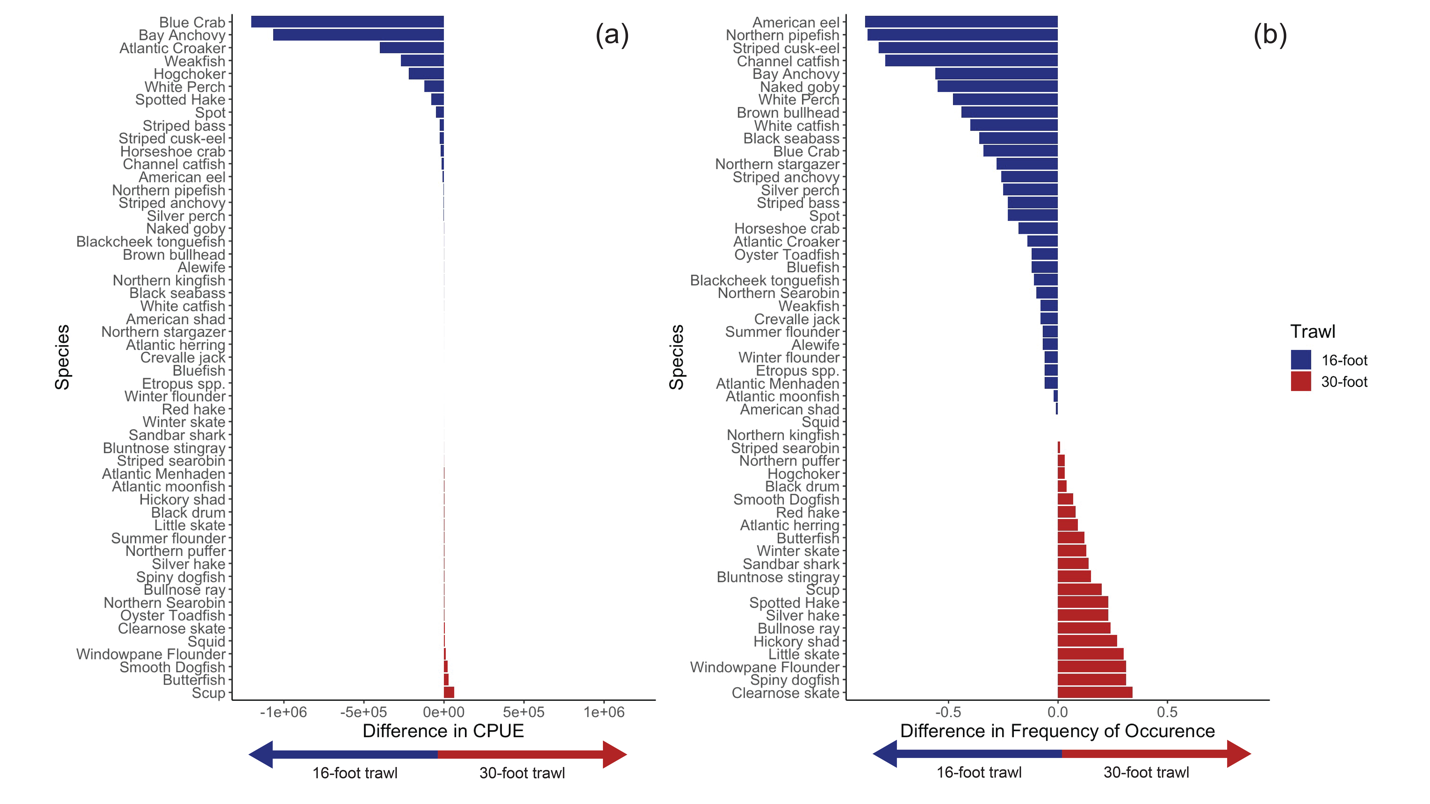


Fig 1


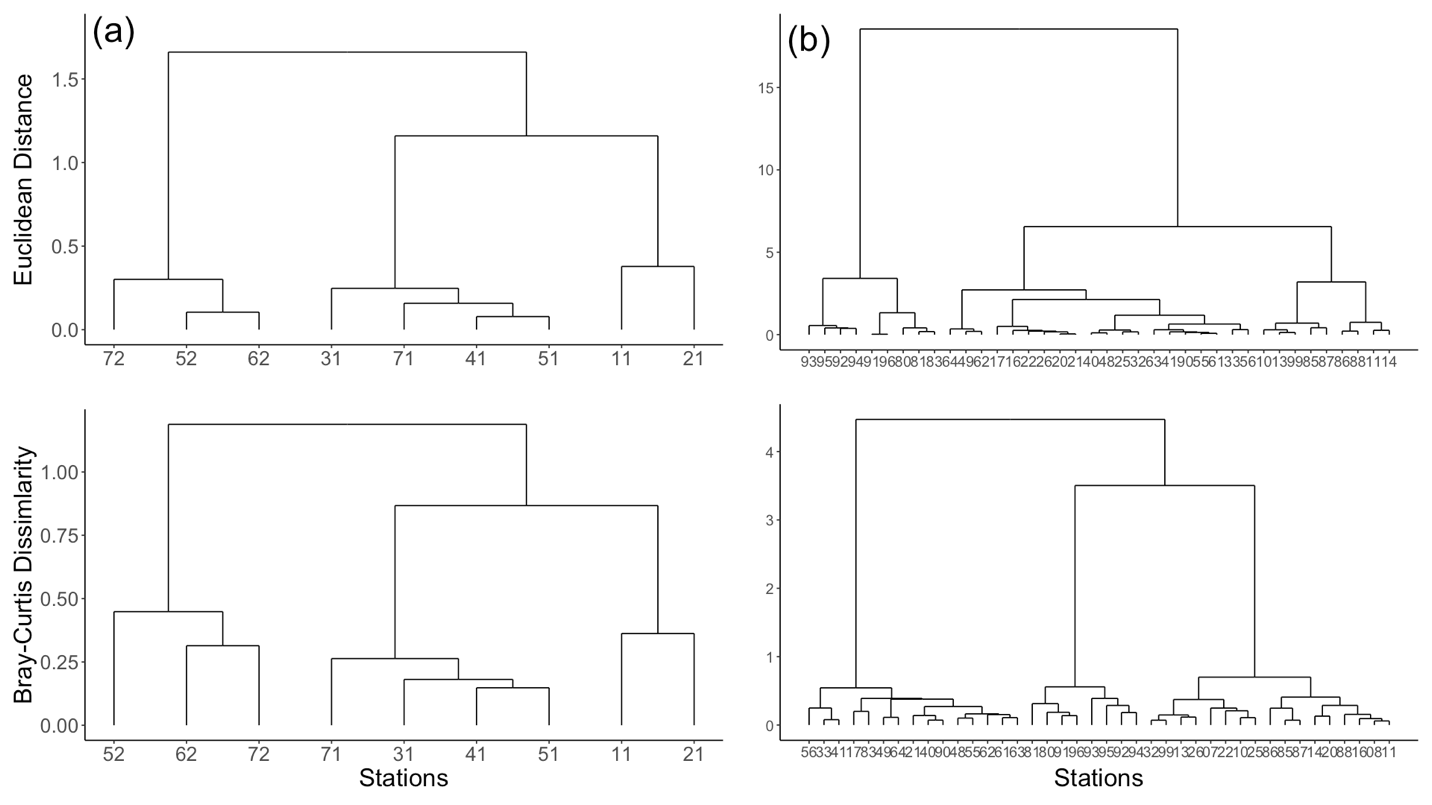


Fig 2


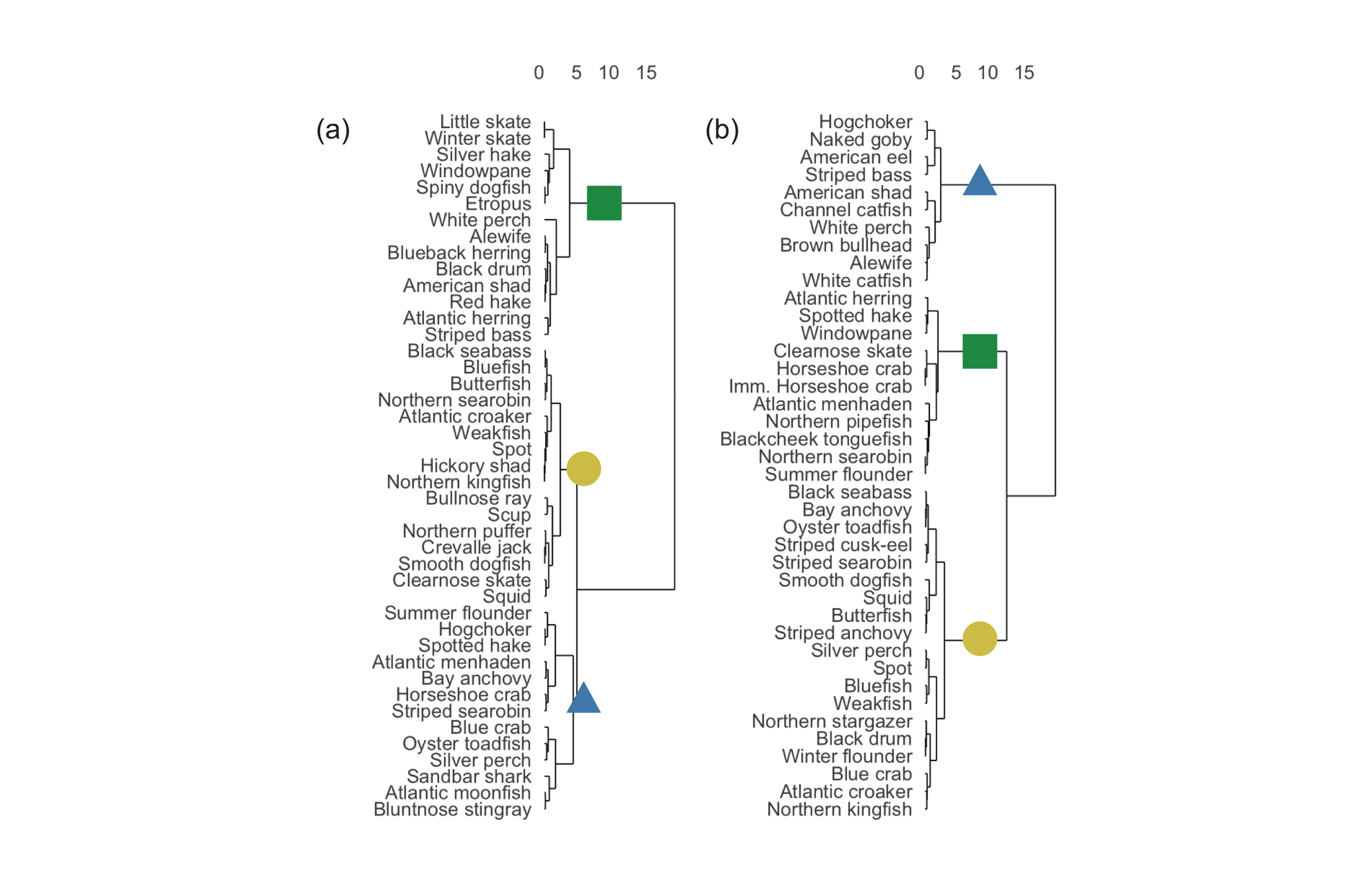


Fig 3


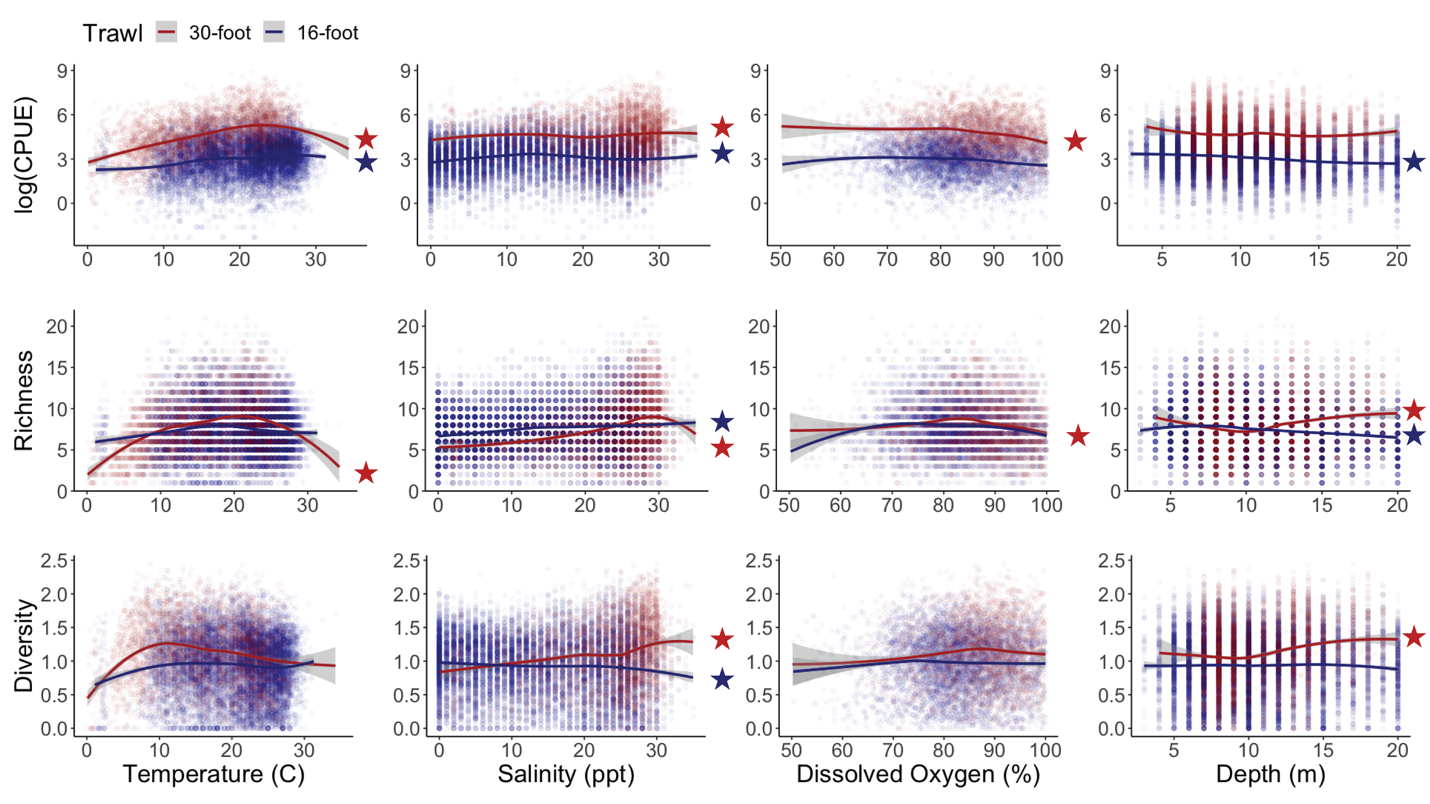


Fig 4


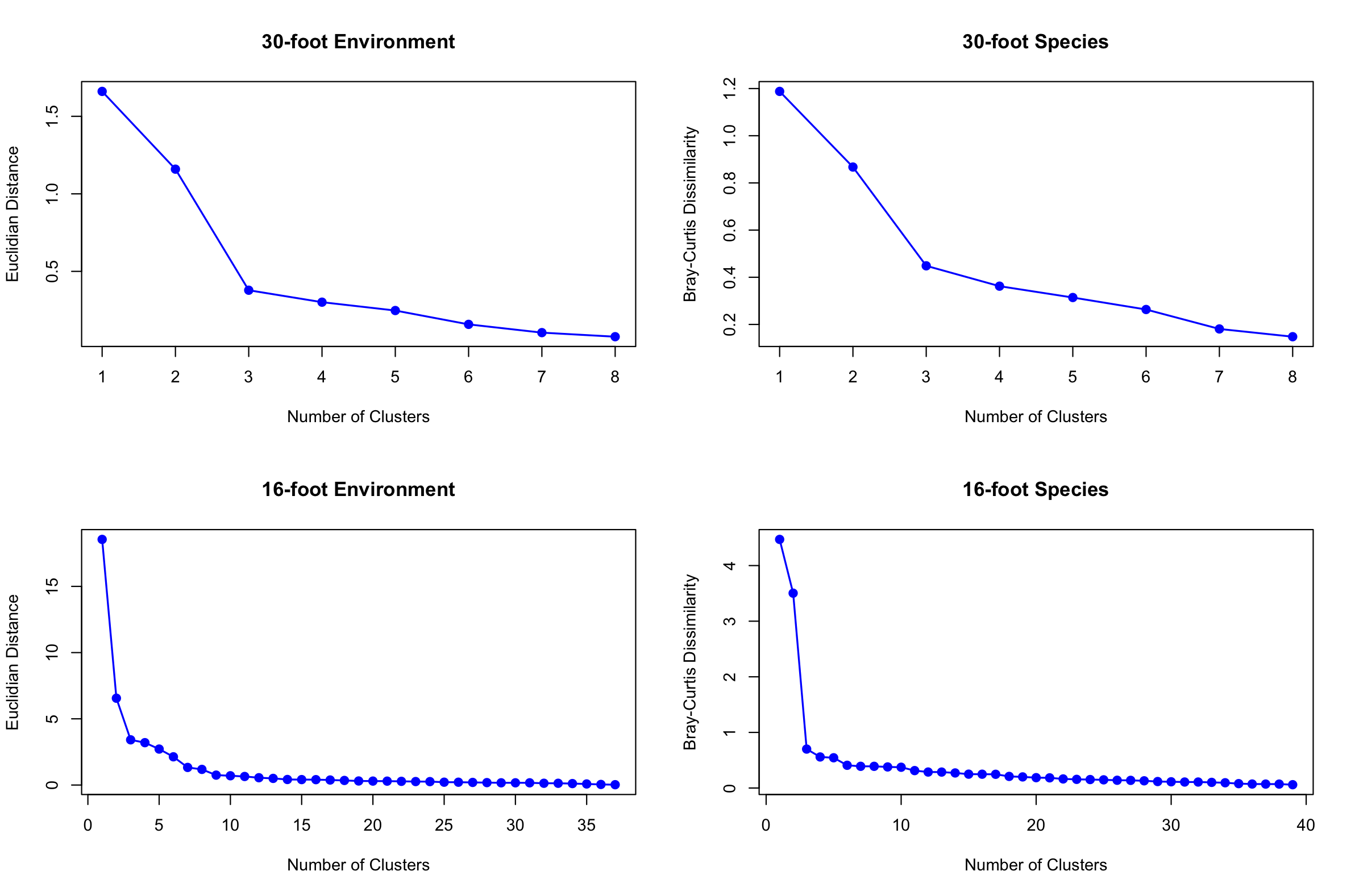


Fig 5

**Table 1.** Overall mean surface and bottom temperature (°C), salinity (parts per thousand), and dissolved oxygen (% saturation) for both surveys across all years and stations sampled.

| ***Variable*** | ***30-foot Survey*** | ***16-foot Survey*** |
| --- | --- | --- |
| Surface Temperature (℃) | 16.5 (± 7.0) | 20.5 (± 5.5) |
| Bottom Temperature (℃) | 16.1 (± 6.7) | 19.5 (± 4.9) |
| Surface Salinity (ppt) | 20.5 (± 7.5) | 14.1 (± 9.0) |
| Bottom Salinity (ppt) | 23.1 (± 7.0) | 15.2 (± 9.3) |
| Surface Dissolved Oxygen (%) | 96.2 (± 14.8) | 83.8 (± 9.2) |
| Bottom Dissolved Oxygen (%) | 90.5 (± 12.9) | 78.33 (± 10.9) |
